# Effect of adjuvanting RBD-dimer-based subunit COVID-19 vaccines with Sepivac SWE™

**DOI:** 10.1016/j.vaccine.2023.03.035

**Published:** 2023-04-24

**Authors:** Senyu Xu, Huixin Duan, Yaling An, Xiyue Jin, Minrun Duan, Patrice M. Dubois, Yan Huang, Kun Xu, Heng Du, Harry Kleanthous, Lianpan Dai, George F. Gao

**Affiliations:** a Savaid Medical School, University of Chinese Academy of Sciences, Beijing, China; b Research Network of Immunity and Health (RNIH), Beijing Institutes of Life Science, Chinese Academy of Sciences, Beijing 100101, China; c CAS Key Laboratory of Pathogen Microbiology and Immunology, Institute of Microbiology, Chinese Academy of Sciences, Beijing, China; d School of Life Sciences, University of Science and Technology of China, Hefei, Anhui 230026, China; e School of Life Sciences, Yunnan University, Kunming 650091, China; f Vaccine Formulation Institute, 1228Plan-Les-Ouates, Geneva, Switzerland; g Beijing Jingdu Children's Hospital, Beijing, China; h Bill & Melinda Gates Foundation, Beijing 100027, China; i Bill & Melinda Gates Foundation, PO Box 23350, Seattle, WA 98102, USA

**Keywords:** COVID-19 vaccine, SARS-CoV-2 vaccine, Protein subunit vaccine, Adjuvant, SWE

## Abstract

Protein subunit vaccines have been widely used to combat infectious diseases, including the current COVID-19 pandemic. Adjuvants play the key role in shaping the quality and magnitude of the immune response to protein and inactivated vaccines. We previously developed a protein subunit COVID-19 vaccine, termed ZF2001, based on an aluminium hydroxide-adjuvanted tandem-repeat dimeric receptor-binding domain (RBD) of the viral spike (S) protein. Here, we described the use of a squalene-based oil-in-water adjuvant, Sepivac SWE™ (abbreviated to SWE), to further improve the immunogenicity of this RBD-dimer-based subunit vaccines. Compared with ZF2001, SWE adjuvant enhanced the antibody and CD4^+^ T-cell responses in mice with at least 10 fold of dose sparing compared with ZF2001 adjuvanted with aluminium hydroxide. SWE-adjuvanted vaccine protected mice against SARS-CoV-2 challenge. To ensure adequate protection against the currently circulating Omicron variant, we evaluated this adjuvant in combination with Delta-Omicron chimeric RBD-dimer. SWE significantly increased antibody responses compared with aluminium hydroxide adjuvant and afforded greater neutralization breadth. These data highlight the advantage of emulsion-based adjuvants to elevate the protective immune response of protein subunit COVID-19 vaccines.

## 1 Introduction

Protein subunit vaccines have been widely used in prophylaxis of infectious diseases with favorable records of safety, efficacy and deployment. Currently, a number of protein subunit vaccines against coronavirus disease 2019 (COVID-19) have been approved globally, which have made important contributions to pandemic control [1], [2], [3]. We previously developed a COVID-19 protein subunit vaccine ZF2001, comprising the dimeric tandem-repeat receptor-binding domain (RBD) of the spike (S) protein from the HB-01 ancestral sequence as an immunogen with aluminium hydroxide as the adjuvant [4], [5]. ZF2001 was safe, well-tolerated and immunogenic in human, with a three-dose vaccination regimen (25 μg/dose) [6]. In Phase 3 clinical trial, vaccine efficacy was 81.4 % in preventing COVID-19 of any severity [7]. ZF2001-elicited neutralizing activities were largely preserved against pseudoviruses expressing S protein from SARS-CoV-2 variants, including Alpha (B.1.1.7), Beta (B.1.351), Gamma (P.1) and Delta (B.1.617.2), but were declined substantially against the currently circulating Omicron (B.1.1.529) and its sub-variants [8], [9]. To better combat the Omicron variant, we had previously developed a multivalent chimeric RBD-dimer, which was demonstrated to elicit broader serum neutralization of SARS-CoV-2 variants compared with the monovalent RBD homodimers [10].

For protein subunit vaccines, adjuvant helps to improve the quality and magnitude of the vaccine-induced immune response [11]. Aluminium hydroxide is the most widely used adjuvant, with billions of doses used in vaccines against several infectious disease targets. Globally, a number of aluminium hydroxide-adjuvanted COVID-19 vaccines have been deployed in the vaccination campaign against this pandemic (https://covid19.trackvaccines.org/). However, other adjuvants developed during recent decades have been shown to induce more potent immune responses compared with aluminium hydroxide, such as AS03 and MF59 used in pandemic SARS-CoV-2 vaccines [11] or influenza vaccines [12], [13], [14], [15]. Therefore, to better control the Omicron surge, development of next generation COVID-19 vaccines using more potent adjuvants with variant-adapted immunogens would enhance the vaccine efficacy and help control the spread of variants arising from the current pandemic. To date, several non-aluminium hydroxide adjuvanted vaccines have been used in the COVID-19 responses, including emulsions (AS03), saponin-based (Matrix-M), and TLR-agonists (CpG1018 and TLR7/8 ligand) [16]. Sepivac SWE™ (abbreviated to SWE) is a squalene-based oil-in-water emulsion adjuvant [17]. Squalene-based emulsion adjuvants such as MF59 have an efficacy and safety profile well-documented in seasonal and pandemic influenza vaccines [18]. Squalene-based oil-in-water adjuvant AS03 has been approved in COVID-19 vaccine COVLP [19] and VidPrevtyn Beta (https://www.ema.europa.eu/en/documents/product-information/vidprevtyn-beta-epar-product-information_en.pdf, https://www.ivi.int/sk-biosciences-covid-19-vaccine-approved-for-use-by-republic-of-korea/). Therefore, we sought to further improve our RBD-dimer-based protein subunit COVID-19 vaccine through use of the SWE adjuvant.

In this study, we evaluated the effect of SWE in adjuvanting the RBD-dimer immunogen in mice. SWE largely improved both the humoral and cellular immune responses of the vaccine compared with the licensed aluminium hydroxide-adjuvanted vaccine, with 10-times dose sparing observed. Low doses of vaccine were shown to confer protection in mice against SARS-CoV-2 challenge when formulated with a novel bivalent Delta-Omicron RBD-dimer immunogen which was designed to control SARS-CoV-2 variants. SWE was superior to aluminium hydroxide in eliciting elevated and broad neutralizing antibodies against SARS-CoV-2, including all Omicron sub-variants. Our data support using novel adjuvants, like SWE, with variant-adapted multivalent immunogens to control SARS-CoV-2 viral evolution through upgrading of the COVID-19 vaccine.

## 2 Results

### 2.1 SWE adjuvanted RBD-dimer elicits robust VN antibody responses

**Fig. 1:**
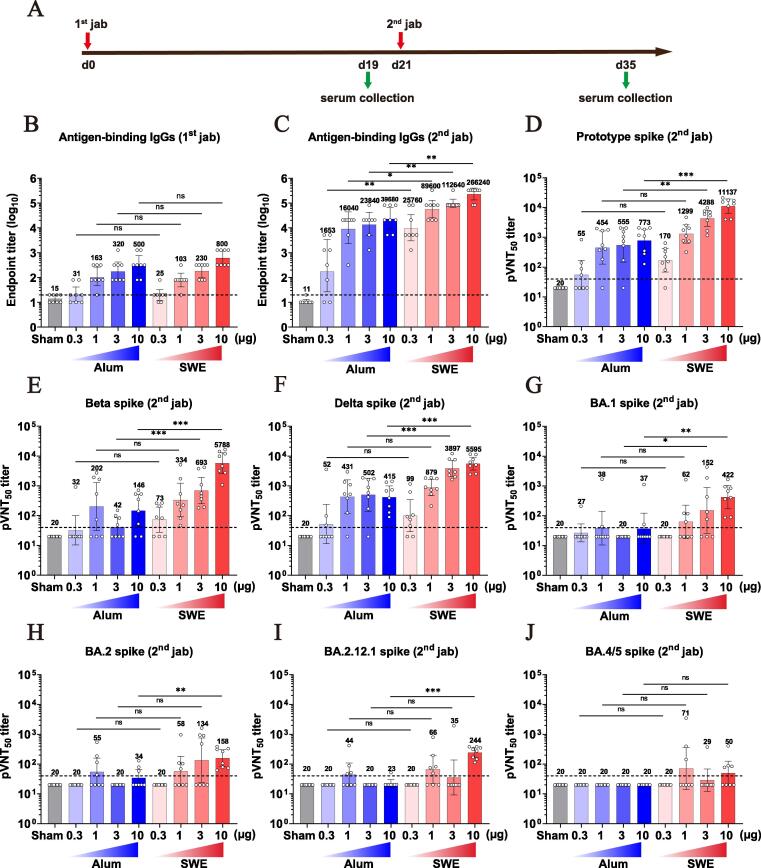
**Prototype RBD-dimer with Sepivac SWE induced robust humoral immune responses.** Eight groups of 8–9 weeks-old female BALB/c mice (n = 8, each group) were immunized with two doses of prototype RBD-dimer with escalation dose of 0.3 μg, 1 μg, 3 μg and 10 μg formulated with Sepivac SWE (red) or aluminium hydroxide (blue), 21 days apart. PBS alone was given as the sham control. The doses shown in figures all indicate those of antigen. P-values were analyzed with two-tailed Mann-Whitney test (ns, P > 0.05; * P < 0.05; ** P < 0.01; *** P < 0.001). A) Schedule of immunization and sampling. (B, C) Prototype RBD-specific IgG titres in sera tested by ELISA after once (B) or twice (C) immunization. (D, E, F, G) Sera collected at 14 days post the second immunization were tested for neutralization of a panel of pseudotyped viruses displaying prototype, Beta, Delta, BA.1, BA.2, BA.2.12.1 and BA.4/5 spike. The values of 50 % pseudovirus neutralization titer (pVNT_50_) are shown as the GMT with 95 % CI. The horizontal dashed line indicates the limit of detection (LOD).

We performed a head-to-head comparison of our prototype RBD dimer vaccine to evaluate immunogenicity using either SWE or aluminium hydroxide adjuvant. Groups of BALB/c mice were vaccinated with two doses of SWE-adjuvanted or aluminium hydroxide-adjuvanted prototype RBD-dimer in a dose escalation manner (antigen: 0.3 μg, 1 μg, 3 μg and 10 μg per dose), 21 days apart (Fig. 1**A**). A group receiving PBS was used as the sham control. Two weeks post the second immunization, the mice sera were collected to detect prototype antigen-binding IgGs and neutralizing antibodies against pseudovirus displaying spike protein from the prototype SARS-CoV-2 or the representative variants of concern (VOCs), including Beta, Delta and Omicron (BA.1, BA.2, BA.2.12.1 and BA.4/5 sub-variants).

The RBD-binding IgGs were induced in a dose-dependent manner for vaccines adjuvanted with either the SWE or aluminium hydroxide after both the priming and boosting vaccinations (Fig. 1**B and C**). After priming, the endpoint IgG geometric mean titers (GMTs) were between 31 (0.3 µg dose) and 500 (10 µg dose) in the aluminium hydroxide group, and between 25 (0.3 µg dose) and 800 (10 µg dose) in the SWE group (Fig. 1**B**). After boosting, differences of antibody responses between groups receiving different adjuvants widened. The GMTs were between 1653 (0.3 µg dose) and 39,680 (10 µg dose) in the aluminium hydroxide group, and significantly enhanced to between 25,760 (0.3 µg dose) and 266,240 (10 µg dose) for vaccine adjuvanted with SWE (Fig. 1**C**). These results indicate that SWE elicits strong antibody responses and had a notable dose-sparing effect.

We next evaluated the serum neutralization of pseudotyped viruses displaying S protein from the prototype SARS-CoV-2, or VOCs including Beta, Delta and Omicron (BA.1, BA.2, BA.2.12.1 and BA.4/5 sub-variants) (Fig. 1**D-J**). Consistent with the trends found in the binding antibodies, both adjuvants elicited neutralizing antibodies in a dose-dependent manner. In the aluminium hydroxide group, the GMTs were between 55 and 773 against the prototype pseudovirus, and declined to between 52 and 415 against the Delta variant, and further declined to between 32 and 202 against Beta, and were almost below the limit of detection (LOD) against all evaluated Omicron sub-variants (Fig. 1**D-J**). By contrast, SWE elicited much higher neutralizing antibodies, with GMTs between 170 and 11,137 against the prototype pseudovirus, between 99 and 5595 against Delta, and between 73 and 5788 against Beta (Fig. 1**D-F**). Notably, a higher dose (10 µg) of SWE-adjuvanted vaccine elicited 100 % seroconversion of neutralizing antibody against several Omicron sub-variants in mice, with documented GMT of 422 against BA.1, 158 against BA.2, 244 against BA.2.12.1 (Fig. 1**G-I**). In addition, two doses of SWE-adjuvanted vaccine could still elicit neutralizing antibodies against BA.4/5, with the GMTs between 29 and 71 (Fig. 1**J**). A 1 µg dose of immunogen adjuvanted with SWE was also shown to elicit superior neutralizing antibodies compared with 10 µg dose of the approved ZF2001 vaccine (adjuvanted with aluminium hydroxide) and with>10 times dose sparing potential.

### 2.2 SWE-adjuvanted RBD-dimer elicits detectable cellular responses

**Fig. 2:**
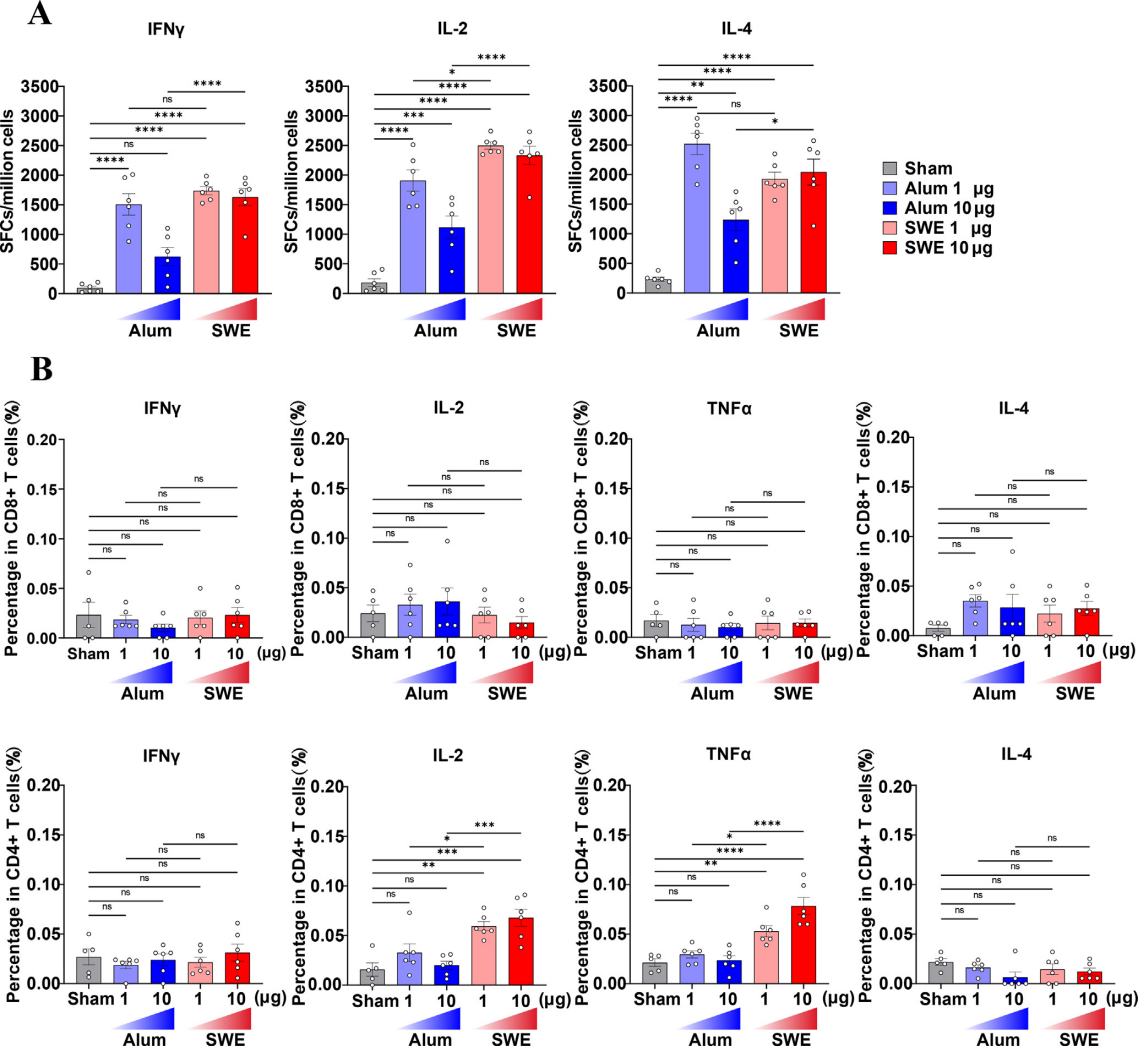
**Cellular immune responses at 14 days after the second vaccination.**(A) Cellular immune responses tested by IFNγ, IL-2 and IL-4 ELISPOT. SFCs: Spot-forming cells.(B) RBD-specific CD8^+^ and CD4^+^ T-cell responses quantified by flow cytometry after *in vitro* stimulation with peptide pool spanning the SARS-CoV-2 RBD. Expression of interferon gamma (IFNγ), interleukin (IL)-2, tumor necrosis factor (TNF)α and interleukin (IL)-4 in CD8^+^ and CD4^+^ T cells were showed respectively. The values showed in (A) and (B) were the means with SEM. P-values were analyzed with one-way ANOVA with multiple comparisons (ns, P > 0.05; * P < 0.05; ** P < 0.01; *** P < 0.001; **** P < 0.0001).

To explore the T cell responses induced by the adjuvanted COVID-19 vaccines, we performed enzyme-linked immunospot assay (ELISPOT) and intracellular cytokine staining (ICS) to measure the splenocyte cytokine production. Prototype RBD-dimer formulated with either aluminium hydroxide or SWE induced substantial RBD-specific cellular responses upon *in vitro* stimulation on day 14 after boosting, with balanced secretion of both Th1 (IFNγ, IL-2) and Th2 (IL-4) cytokines detected by ELISPOT (Fig. 2**A**). Notably, SWE elicited significantly higher production of IFNγ and IL-2 in comparison to aluminium hydroxide, in particular, at the 10 µg dose (Fig. 2**A**). Furthermore, SWE was found to induce CD4^+^ T cell responses with measurable induction of Th1 cytokines (IL-2 and TNFα) (Fig. 2**B**). These results support that, compared with aluminium hydroxide adjuvant, SWE elicits apparently stronger T-cell responses.

### 2.3 Protective efficacy of SWE-adjuvanted RBD-dimer vaccine

**Fig. 3:**
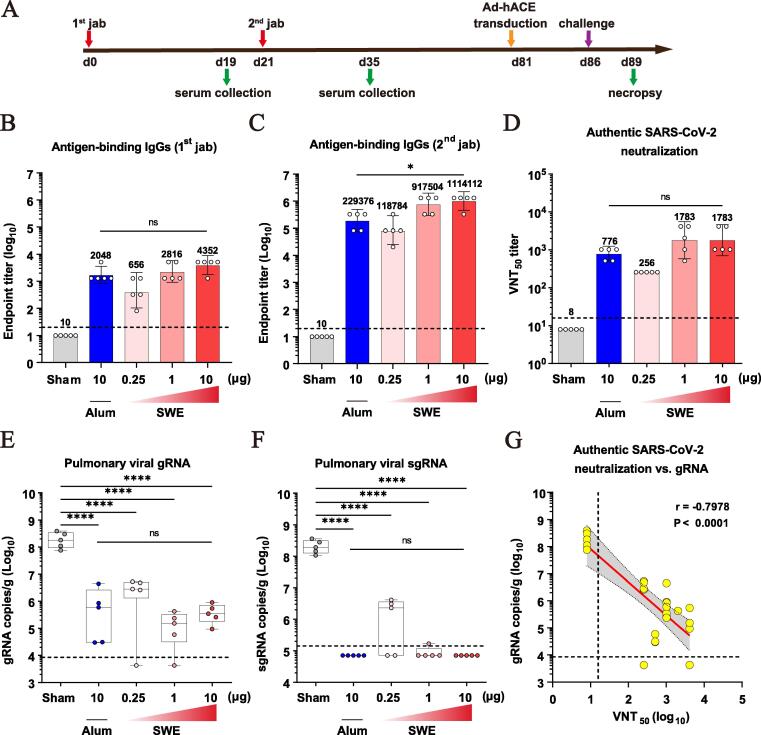
**Protective efficacy of prototype RBD-dimer with Sepivac SWE.**Four groups of 6–8 weeks-old female BALB/c mice (n = 5, each group) were immunized two doses of prototype RBD-dimer with escalation dose of 0.25 μg, 1 μg, 10 μg formulated with Sepivac SWE (red) and 10 μg ZF2001 including aluminium hydroxide (blue), 21 days apart. PBS alone was given as the sham control. Then, mice were transduced with 8 × 10^9^ vp of Ad5-hACE2 via intranasal route. Five days later, five groups of mice (n = 25) were intranasally challenged with 5 × 10^5^ TCID_50_ of prototype SARS-CoV-2. Mice were euthanized and necropsied at 3 DPI. The doses shown in figures all indicate those of antigen.(A) Schedule of immunization, sampling and viral challenge. (B, C) Prototype RBD-specific IgG titres in sera tested by ELISA after once (B) or twice (C) immunization. The horizontal dashed line indicates the LOD. (D) 50 % neutralization titer of authentic prototype SARS-CoV-2 in sera of 14 days post the second jab. The values shown in (B)–(D) are the GMT with 95 % CI. (E, F) Box and whiskers plots of 25th to 75th percentile with median as center and whiskers of minimum to maximum percentile prototype viral gRNA and sgRNA in lung tissues at 3 DPI. P-values were analyzed with one-way ANOVA with multiple comparisons (ns, P > 0.05; * P < 0.05; ** P < 0.01; *** P < 0.001; **** P < 0.0001) (G) Plots show correlation and corresponding two-sided P value between pVNT_50_ and viral gRNA copies of prototype SARS-CoV-2.

To further evaluate the protective efficacy of vaccine formulated with SWE *in vivo*, groups of female BALB/c mice (n = 5) were immunized with two doses of SWE-adjuvanted vaccine, given as graded doses (antigen: 0.25 μg, 1 μg or 10 μg), 21 days apart (Fig. 3**A**). A 10 μg dose of ZF2001 (aluminium hydroxide adjuvanted) and PBS were given as controls. Vaccines administered with either aluminium hydroxide or SWE as the adjuvant elicited high levels of both RBD-binding IgGs and neutralizing antibodies against authentic prototype SARS-CoV-2 (Fig. 3**B-3D**). Consistent with the immunogenicity evaluation shown before, a 1 µg dose of SWE-adjuvanted vaccine was able to induce higher binding and neutralizing antibodies compared with a 10 µg dose of ZF2001 vaccine using aluminium hydroxide as the adjuvant (Fig. 3**B-D**).

Two months post booster vaccination, 8 × 10^9^ viral particles of adenovirus expressing hACE2 was transduced into the immunized mice via the intranasal route to establish a SARS-CoV-2-sensitive challenge model [20]. Five days later, transduced mice were intranasally challenged with 5 × 10^5^ 50 % tissue culture infectious dose (TCID_50_) of SARS-CoV-2 (hCoV-19/China/CAS-B001/2020, GISAID No. EPI_ISL_514256-7) [21], [22]. Mice were euthanized and necropsied at 3 days post infection (DPI) to detect viral loads in lungs. The mean titers of viral genomic RNA (gRNA) per gram of lung were reduced > 2 logs in both the aluminium hydroxide group (10 µg dose) and the SWE groups (both 1 and 10 µg dose) (Fig. 3**E**). Neutralizing antibody titers inversely correlated with the copies of pulmonary gRNA of prototype SARS-CoV-2 (r = −0.7978, P < 0001) based on a linear model (Fig. 3**G**). To preclude the possibly gRNA derived from the input challenge viruses, we quantified subgenomic RNA (sgRNA) of the *E* gene [23] to detect the replicating virus [24]. In line with the trends in neutralizing antibody responses, 3 out of 5 mice in the 0.25 µg dose group with SWE adjuvant exhibited substantial levels of pulmonary viral sgRNA. By contrast, mice receiving either the 10 µg dose of ZF2001 or 1 or 10 µg dose of SWE-adjuvanted vaccine showed almost undetectable viral sgRNA in lungs (Fig. 3**F**).

### 2.4 SWE adjuvant expands breadth of neutralization when co-administered with updated Delta-Omicron RBD-dimer vaccine

**Fig. 4:**
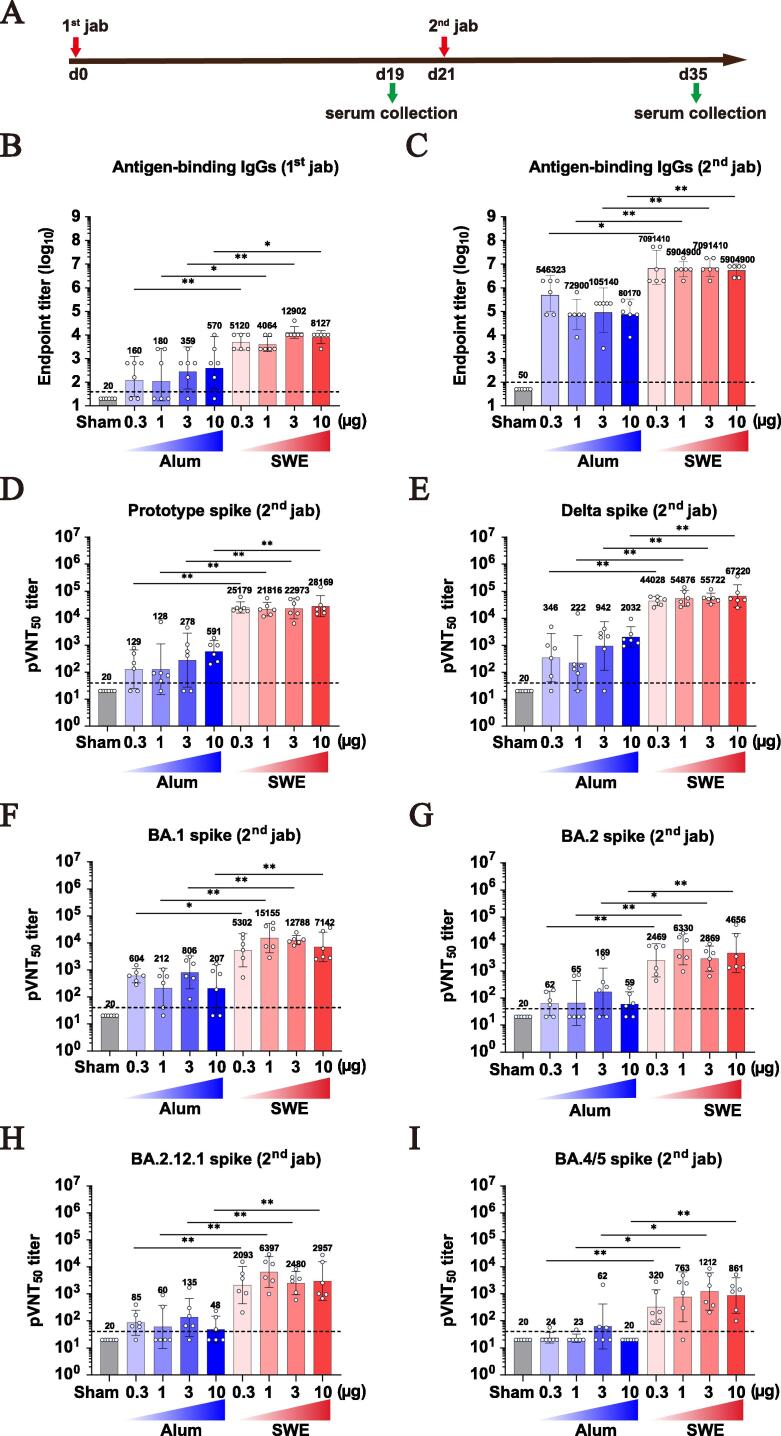
**Delta-Omicron chimeric RBD-dimer formulated withSWE elicited stronger humoral immune responses.** Eight groups of 6–8 weeks-old female BALB/c mice (n = 6, each group) were immunized with two doses of Delta-Omicron RBD-dimer with escalation dose of 0.3 μg, 1 μg, 3 μg and 10 μg formulated with SWE (red) or aluminium hydroxide (blue), respectively. PBS alone was given as the sham control. The doses shown in figures all indicate those of antigen. P-values were analyzed with two-tailed Mann-Whitney test.(A) Schedule of immunization and sampling. (B, C) Delta-Omicron RBD-dimer-specific IgG titres in sera following once (B) and twice (C) immunization with Delta-Omicron chimeric RBD-dimer formulated with the indicated adjuvants were tested by ELISA. The antigen used for ELISA in Fig. 4B-C is Delta-Omicron RBD-dimer protein. (D, E, F, G, H, I) Neutralizing antibody levels against SARS-CoV-2 pseudovirus of prototype, Delta, BA.1, BA.2, BA.2.12.1 and BA.4/5 variants post the second vaccination. The horizontal dashed line indicates the limit of detection. The values shown in (B)-(I) are the GMT with 95 % CI.

To adapt to emerging SARS-CoV-2 viral variants, we recently described generation of an improved, chimeric RBD-dimer approach, designed to elicit broader responses against both circulating and emerging variants [10]. A chimeric Delta-Omicron RBD-dimer immunogen was shown to stimulate broader and more balanced neutralizing antibodies across the different variants, compared with the homotypic prototype RBD-dimer [10]. Therefore, we next evaluated whether SWE-adjuvanted Delta-Omicron RBD-dimer immunogen can further improve the vaccine potency and breadth of response against multiple variants. After priming, the binding antibody elicited by SWE-adjuvanted vaccine increased up to 36 fold compared with the aluminium hydroxide group (Fig. 4**B**). After boosting, differences between the two adjuvants groups were further widened. The GMT of binding antibodies in the SWE group increased up to 81 fold compared with the aluminium hydroxide group (Fig. 4**C**). Additionally, when compared with aluminium hydroxide, the SWE immunized animals showed significant improvement of vaccine-elicited serum neutralization of multiple SARS-CoV-2 pseudoviruses, including the prototype, Delta, and all Omicron sub-variants (BA.1, BA.2, BA.2.12.1, BA.4/5). GMTs were increased between 48 and 195-fold against the prototype (Fig. 4**D**), between 33 and 247-fold against Delta (Fig. 4**E**), between 9 and 91-fold against BA.1 (Fig. 4**F**), between 17 and 97 to BA.2 (Fig. 4**G**), between 18 and 107-fold against BA.2.12.1 (Fig. 4**H**), and between 13 and 43-fold against BA.4/5 (Fig. 4**I**). Furthermore, compared with the response following immunization with the prototype SARS-CoV-2 vaccine, SWE significantly enhanced antibody levels for the Delta-Omicron RBD-dimer immunogen. Even at the lowest antigen dose (0.3 μg), SWE-adjuvanted vaccine induced much higher neutralizing antibodies than at the highest dose (10 μg dose) of an equivalent aluminium hydroxide-adjuvanted vaccine, with>33-fold of dose-sparing, and an ∼ 10–200-fold neutralizing antibody improvement for the Delta-Omicron RBD-dimer immunogen.

### 2.5 SWE-adjuvanted Delta-Omicron RBD-dimer induces substantial cellular responses

**Fig. 5:**
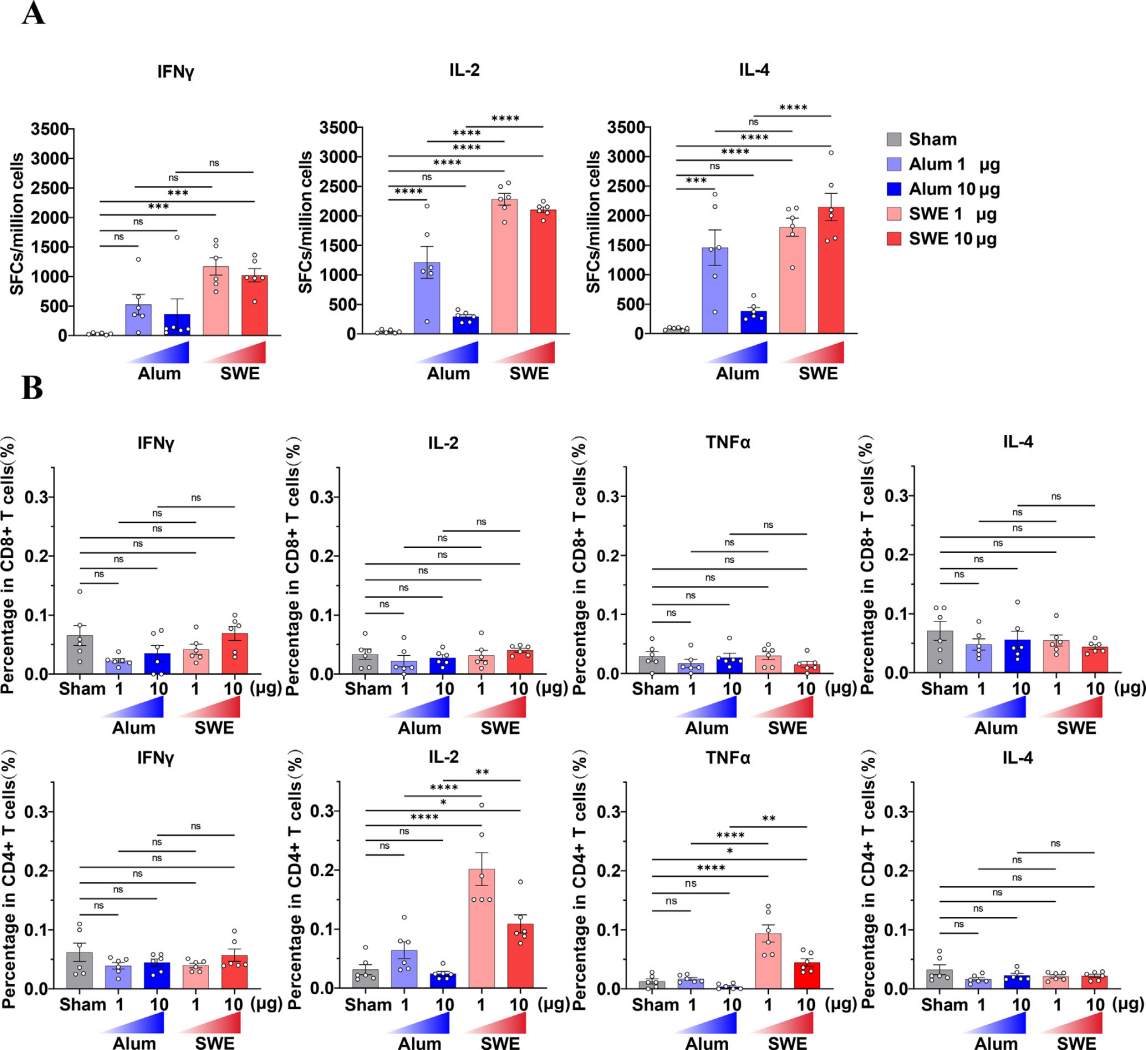
**Cellular immune responses at 14 days after the second vaccination.** (A) Cellular immune responses tested by IFNγ, IL-2 and IL-4 ELISPOT. SFCs: Spot-forming cells. (B) RBD-specific CD8^+^ and CD4^+^ T-cell responses quantified by flow cytometry after *in vitro* stimulation with peptide pool spanning the SARS-CoV-2 RBD. Expression of IFNγ, IL-2, TNFα and IL-4 in CD8^+^ and CD4^+^ T cells are showed respectively. The values showed in (A) and (B) are the means with SEM. P-values were analyzed with one-way ANOVA with multiple comparisons (ns, P > 0.05; * P < 0.05; ** P < 0.01; *** P < 0.001; **** P < 0.0001).

Either SWE or aluminium hydroxide induced both Th1 and Th2 cytokine production (Fig. 5A). Compared with aluminium hydroxide-adjuvanted group, SWE-adjuvanted Delta-Omicron chimeric RBD-dimer vaccine can induce a stronger Th1 cell immune responses, with more production of IL-2 and TNFα, a trend similar to the prototype SARS-CoV-2 RBD-dimer vaccines (Fig. 5B).

## 3 Discussion

It has been reported that squalene-based oil-in-water adjuvants can enhance vaccine immunogenicity and spare antigen dose when formulated with H5N1 influenza vaccines [25]. In the late 1990s, MF59, a squalene-based oil-in-water adjuvant, was the first adjuvant included in licensed vaccines since aluminium hydroxide [16], demonstrating enhanced antibody responses when formulated with the influenza vaccines Fluad® [26]. In addition, it was reported that MF59 not only improved protection but also induced cross-protective neutralizing antibodies compared with aluminium hydroxide adjuvant [27]. Likewise, SWE adjuvant, was also demonstrated to enhance functional antibody responses in ferrets, with similar dose-sparing for an H7N9 split influenza vaccine [18]. SWE, as a squalene-based oil-in-water adjuvant with different formulation, can provide more commercial availability to meet the demands in future for new generation of adjuvants. The SWE-adjuvanted COVID-19 vaccine COVAC-2 has entered into clinical trials (NCT04702178 and NCT05209009).

SWE was previously characterized to be able to elicit humoral and cellular responses [18]. From our study, we found SWE elicited a balanced Th1/Th2 responses when formulated with homotypic or heterotypic RBD-dimer (Fig. 2, Fig. 5). Impressively, compared with aluminium hydroxide adjuvant, SWE induced a significantly stronger Th1 response (IL-2 and TNFα production), suggesting a benefit of SWE adjuvant to elicit cellular responses.

In this study, we demonstrated that SWE adjuvant can largely enhance both the humoral and cellular immune responses for SARS-CoV-2 RBD-dimer immunogens when compared with aluminium hydroxide adjuvant. For the approved SARS-CoV-2 protein subunit vaccine ZF2001 that based on the aluminium hydroxide-adjuvanted prototype RBD-dimer, our data suggests that using SWE as the adjuvant of choice would potentially improve vaccine efficacy and spare immunogen dose. For the Omicron-adapted chimeric RBD-dimer vaccine, SWE showed superiority over aluminium hydroxide adjuvant in immunogenicity, breadth of response and dose-sparing potential. A negative correlation is found between antigen-binding IgGs and pulmonary viral gRNA (Fig. S2A). This antigen-binding IgGs are positively correlated with vaccine dose (Fig. S2B). Therefore, the vaccine dose is positively correlated with the protection. SWE showed a ∼ 10-times dose sparing compared with aluminium hydroxide when the same antigen-binding IgGs are elicited.

A limitation of this study is that the head-to-head adjuvant comparison was performed in a BALB/c mouse model, which has been associated with a reduced ability to express the Th1 cytokine IL-12, and subsequent lower levels of IFNγ [28]. Given the difference in response between mouse and human, we need to further verify the effect of SWE adjuvant in humans for a more relevant evaluation. Given that many people in the world have been administrated COVID-19 vaccines, it would be important to further explore the effect of an SWE adjuvanted vaccine as part of a booster approach with a pan-coronavirus vaccine candidate. Due to the instability of Omicron RBD homodimer protein (prone to form aggregation), we cannot obtain high-quality Omicron RBD-dimer protein. Therefore, we did not compare the admixture of two homotypic RBD-dimer vs. the chimeric RBD-dimer. Besides, due to the space limitation for the biosafety level 3 facilities here, only 10 μg group of aluminium hydroxide-adjuvanted mice were tested for challenge experiment. Therefore, we cannot conclude a greater protective efficacy of SWE over aluminium hydroxide.

In conclusion, SWE allows for dose sparing when formulated with a prototype COVID-19 protein-based vaccine, and it can enhance the breadth of neutralizing antibodies and induces balanced Th1/Th2 immune responses. It is a promising adjuvant when considering updating current subunit COVID-19 vaccines and may have a significant role to play in next generation, multivalent variant-adapted vaccines.

## 4 Materials and methods

### 4.1 Cells and viruses

Human embryonic kidney cells 293T (HEK293T cells) (ATCC CRL-3216), African green monkey kidney epithelial cells (Vero cells) (ATCC CCL81), Baby hamster syrian kidney cells (BHK-21 cells) (ATCC CCL-10) and Vero E6 cells (ATCC CRL-1586) were maintained in Dulbecco’s modified Eagle’s medium (DMEM, Invitrogen, USA) supplemented with 10 % fetal bovine serum (FBS) at 37 °C under 5 % CO_2_. SARS-CoV-2 (hCoV-19/China/CAS-B001/2020, GISAID No. EPI_ISL_514256-7) was propagated in Vero-E6 cells.

### 4.2 Vaccines and adjuvants

Protein subunit vaccine ZF2001 were manufactured according to good manufacturing practice guidelines by Anhui Zhifei Longcom Biopharmaceutical Co. Ltd. The vaccine contains SARS-CoV-2 tandem-repeat dimeric form of RBD antigen (S protein 319-537, GISAID accession No. EPI_ISL_402119) manufactured in the CHO cell line, with aluminium hydroxide as the adjuvant.

The adjuvant Sepivac SWE™ is a squalene-based oil-in-water adjuvant, comprising squalene (3.9 %, w/v), sorbitan trioleate (0.47 %, w/v), and polyoxyethylene (80) sorbitan monooleate (0.47 %, w/v) dispersed in 10 mM citrate buffer at pH 6.5 [18]. Sepivac SWE™ was developed by the Vaccine Formulation Institute (Switzerland) and Seppic (France) and is available at GMP grade under an open access model [17].

For vaccines preparation in various doses, we mixed equal volume of antigen with SWE. The terminal concentration of aluminium hydroxide is 0.5 mg/mL. The doses shown in figures all indicate those of antigens.

### 4.3 Mice

Specific pathogen-free (SPF) 6–8-week old female BALB/c mice were purchased from Beijing Vital River Laboratory Animal Technology Co., Ltd. (licensed by Charles River) and housed under SPF conditions in the laboratory animal facilities at Institute of Microbiology, Chinese Academy of Sciences (IMCAS). Mice were housed with 4 companions per cage. All mice were allowed free access to water and standard chow diet and provided with a 12-hour light and dark cycle (temperature: 20–25 °C, humidity: 40–70 %). All animal experiments were approved by the Committee on the Ethics of Animal Experiments of the IMCAS, and conducted in compliance with the recommendations in the Guide for the Care and Use of Laboratory Animals of the IMCAS Ethics Committee.

### 4.4 Protein expression and purification

The SARS-CoV-2 monomeric RBD (S protein 319-541, GISAID accession No. EPI_ISL_402119) was used in ELISA. Prototype SARS-CoV-2 RBD-dimer was two RBD (GenBank: YP_009724390, S protein residues 319–537) connected as tandem repeat. Delta-Omicron chimeric SARS-CoV-2 RBD-dimer was one Delta RBD (S protein residues 319–537) and one Omicron BA.1 RBD (S protein residues 316–534) connected as tandem repeat. There was no linker between either of two tandem RBD-dimers. For each construct, signal peptide sequence of MERS-CoV S protein (S protein residues 1–17) was added to the protein N terminus for protein secretion, and a hexa His tag was added to the C terminus to facilitate further purification processes. These constructs were codon-optimized for mammalian cell expression and synthesized by GENEWIZ, China.

Prototype SARS-CoV-2 monomeric RBD protein, prototype SARS-CoV-2 RBD-dimer and Delta-Omicron chimeric RBD-dimer were expressed and purified as previously described [4], [10]. Briefly, the constructs were transiently transfected into Expi293F™ cells. After 5 days, the supernatant was collected and soluble protein was purified by Ni affinity chromatography using a HisTrap™ EXCEL 5 mL column (GE Healthcare). The sample was further purified via gel filtration chromatography with HiLoad® 16/600 Superdex® 200 pg (GE Healthcare) in a buffer composed of 20 mM Tris-HCl and 150 mM NaCl (pH 8.0). The eluted peaks were analyzed by SDS-PAGE for protein size and purity.

### 4.5 Mouse experiments

For immunization of mice, four different doses of prototype SARS-CoV-2 RBD-dimer or Delta-Omicron RBD-dimer were respectively diluted and mixed with an equal volume adjuvant Sepivac SWE™（SEPPIC, France） or aluminium hydroxide (Anhui Zhifei Longcom Biopharmaceutical Co. Ltd, China). Female BALB/c mice were immunized intramuscularly (i.m) with equal dose of vaccine at day 0 and day 21. The control group of mice were immunized with phosphate buffer saline, without any adjuvant. For the challenge study, female BALB/c mice were immunized with 0.25 μg, 1 μg or 10 μg antigen mixed with SWE or aluminium hydroxide, respectively. Serum samples were collected after vaccination as indicated in figure legends.

For challenge experiment, female BALB/c mice were intranasally (i.n.) transduced with 8 × 10^9^ vp of Ad5-hACE2 as a mouse model for SARS-CoV-2 infection [29]. Five days later, the transduced mice were infected with 5 × 10^5^ TCID_50_ SARS-CoV-2 (hCoV-19/China/CAS-B001/2020, GISAID No. EPI_ISL_514256-7) via intranasal route. The mice were euthanized and necropsied 3 days after SARS-CoV-2 challenge. Lung tissues were collected for virus titration. All mice experiments related with live SARS-CoV-2 were conducted under animal biosafety level 3 (ABSL3) in IMCAS.

### 4.6 Enzyme-linked immunosorbent assay (ELISA)

ELISA plates (3590; Corning, USA) were coated overnight with 3 μg/mL of SARS-CoV-2 monomeric RBD protein in 0.05 M carbonate-bicarbonate buffer, pH 9.6, and blocked in 5 % skim milk in PBS. Serum samples were serially diluted and added to each well, incubated in 37 °C for 2 h, subsequently removed and washed by PBST (1‰ Tween). Plates were incubated with goat anti-mouse IgG-HRP antibody and washed as mentioned above, subsequently developed with 3,3′,5,5′-tetramethylbenzidine (TMB) substrate. Reactions were stopped with 2 M hydrochloric acid, and the absorbance was measured at 450 nm using a microplate reader (PerkinElmer, USA). The endpoint titers were defined as the highest reciprocal dilution of serum to give an absorbance>2.5-fold of the background values. Antibody titer below the limit of detection was determined as half the limit of detection.

### 4.7 Pseudotyped virus neutralization assay

The SARS-CoV-2 prototype and variants pseudoviruses were constructed with a GFP encoding replication-deficient vesicular stomatitis virus (VSV) vector backbone (VSV-ΔG-GFP) as previously described [30]. The codon-optimized SARS-CoV-2 prototype and variants spike protein with an 18 amino acids truncation at the C-terminal were constructed into the pCAGGS vector [31], [32]. The plasmid expressing spike protein was transfected into HEK-293 T cells. VSV-ΔG-GFP pseudovirus were added 24 h after the transfection. Two hours later, the cell culture was removed. DMEM medium supplemented with 10 % FBS and anti-VSV-G antibody (I1HybridomaATCC® CRL2700™) were added. After another 30 h incubation, supernatants were collected, passed through a 0.45 μm filter (Millipore, Cat#SLHP033RB), aliquoted, and stored at −80 °C.

For the neutralization assay, the heat-inactivated serum samples from mice were 2-fold serially diluted (initiating from 1:40) and mixed with equal volume of each pseudovirus at about 1000 transducing units (TU) at 37 °C for 1 h. The mixtures were transferred to pre-plated Vero cells in 96 well plates and incubated at 37 °C for 15 h. After 15 h incubation, the TU numbers were calculated on a CQ1 confocal image cytometer (Yokogawa). Neutralization titer below the limit of detection was determined as half the limit of detection.

### 4.8 Authentic SARS-CoV-2 neutralization assay

The authentic SARS-CoV-2 neutralization assay has been mentioned previously [4]. Briefly, mouse serum samples were 4-fold serially diluted (initiating from 1:16) and mixed with the same volume of SARS-CoV-2 (100 TCID_50_, hCoV-19/China/CAS-B001/2020, GISAID No. EPI_ISL_514256-7), incubated at 37 °C for 1 h. Then, 100 μL virus-serum mixtures were transferred to pre-plated Vero cells in 96-well plates. Inoculated plates were incubated at 37 °C for an additional 72 h to monitor the cytopathic effect (CPE) microscopically. The neutralization titers were defined as the reciprocal of serum dilution required for 50 % neutralization of viral infection. The live virus neutralization assay was conducted under biosafety level 3 (BSL3) facility in IMCAS.

### 4.9 ELISPOT

To detect antigen-specific cellular immune responses, IFNγ, IL-2 and IL-4 based ELISPOT assays were performed. Murine spleens were collected and splenocytes were isolated. Flat-bottom, 96-well plates were pre-coated with 15 μg/mL anti-mouse IFNγ Ab, anti-mouse IL-2 Ab, anti-mouse IL-4 Ab (MABTECH), respectively, overnight at 4 °C and then blocked for 30 min at 37 °C. Murine splenocytes were added to the plate (5 × 10^5^ cells/well). Then, peptides pool (10 μg/mL each) consisting of 20-mers (overlapping by 10 amino acids) spanning the SARS-CoV-2-S RBD or the mixture of Delta-S RBD and Omicron BA.1-S RBD were added to the wells. Phytohemagglutinin (PHA) was added as the positive control. Cells incubated without stimulation were employed as the negative control. After 30 h of incubation, the cells were removed, and the plates were processed in turn with biotinylated detection antibody (MABTECH), streptavidin-HRP conjugate (MABTECH), and BCIP/NBT-plus substrate (MABTECH). When the colored spots were intense enough to be visually observed, the development was stopped by thoroughly rinsing samples with deionized water. The numbers of the spots were determined using an automatic ELISPOT reader.

### 4.10 ICS and flow cytometry

Murine splenocytes were added to the plates (1 × 10^6^ cells/well). Murine splenocytes were stimulated with the peptide pool for 3 h. The cells were then incubated with GolgiStop (BD Biosciences, USA) for an additional 6 h at 37 °C. Then, the cells were harvested and stained with anti-CD3 (BioLegend), anti-CD8α (BioLegend) and anti-CD4 (BioLegend) surface markers. The cells were subsequently fixed and permeabilized (BD Biosciences, USA). Half of the cells were stained with anti-IFNγ (BioLegend), anti-IL-2 (BioLegend) and anti-TNFα (BioLegend). Another half of the cells were stained with anti-IL-4 (Biolegend). All lymphocytes were gated on a LSRFortessa flow cytometer (BD Biosciences, USA) and analyzed with FlowJo software.

### 4.11 Determination of virus titer in lung tissue samples

Mice lung tissues were weighed and homogenized. SARS-CoV-2-specific quantitative Real-time PCR (qRT-PCR) assays were performed using a FastKing One Step Probe RT-qPCR kit (Tiangen Biotech, China) on a CFX96 Touch real-time PCR detection system (Bio-Rad, USA) according to the manufacturer’s protocol. Two sets of primers and probes were used to detect a region of the N gene of viral genome [33] and a region of E gene of subgenomic RNA (sgRNA) [23] from SARS-CoV-2 respectively, with sequences as follows:

gRNA-F, GACCCCAAAATCAGCGAAAT;

gRNA-R, TCTGGTTACTGCCAGTTGAATCTG;

gRNA-probe, FAM-ACCCCGCATTACGTTTGGTGGACC-TAMRA.

(where FAM is 6-carboxyfluorescein, and TAMRA is 6-carboxytetramethylrhodamine);

sgRNA-F, CGATCTCTTGTAGATCTGTTCTC;

sgRNA-R, ATATTGCAGCAGTACGCACACA;

sgRNA-probe, FAM-ACACTAGCCATCCTTACTGCGCTTCG-TAMRA.

Viral loads were expressed on a log_10_ scale as viral copies/gram after calculation with a standard curve. Viral copy numbers below the limit of detection were set as the half of the limit of detection.

## 5 Statistics

The statistical analyses were performed by GraphPad Prism 9.0 for all experiments. Antibody titers were expressed as geometric mean with 95 % CI. Results of ICS and ELISPOT were shown as means with SEM. P-values were analyzed with Mann-Whitney test for ELISA, pseudovirus neutralization and live virus neutralization titers (Fig. 1, Fig. 4). P-values were analyzed with one-way ANOVA with multiple comparisons for viral loads measurements, ELISPOT and ICS assays (Fig. 2, Fig. 5).(ns, P > 0.05; * P < 0.05; ** P < 0.01; *** P < 0.001; **** P < 0.0001).

## Declaration of Competing Interest

Y.A., K.X., L.D., and G.F.G. are listed in the patent as the inventors of the RBD-dimer as a betacoronavirus vaccine. The patent has been licensed to Anhui Zhifei Longcom for protein subunit COVID-19 vaccine development. All other authors declare no competing interests.

## Data Availability

Data will be made available on request.
