# Supplementary figures and images for: Effect of adjuvanting RBD-dimer-based subunit COVID-19 vaccines with Sepivac SWE™
Source: Vaccine. 2023 Apr 24;41(17):2793–803. doi: 10.1016/j.vaccine.2023.03.035 (PMC10028357; doi:10.1016/j.vaccine.2023.03.035)

Figure S1

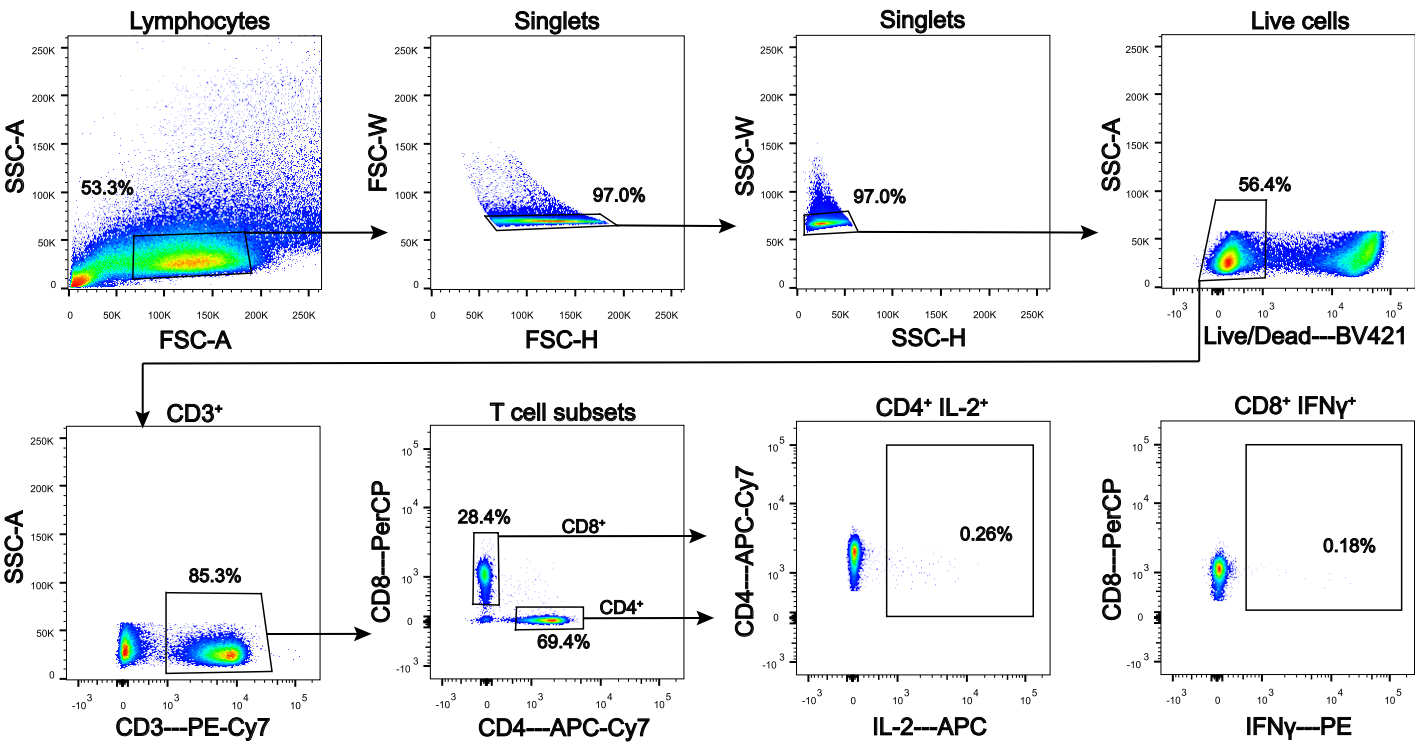

Supplement: Supplementary data 1 [file mmc1.pdf]

Figure S2

A

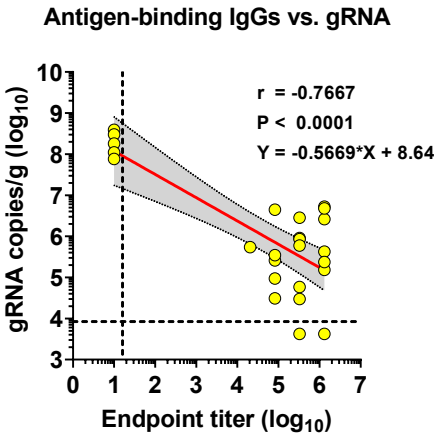

B

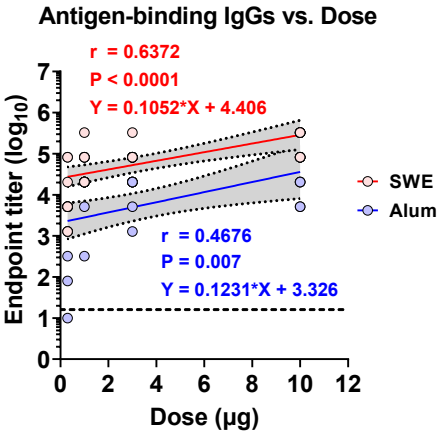

Supplement: Supplementary data 2 [file mmc2.pdf]
